# The illusion of empathy: evaluating AI-generated outputs in moments that matter

**DOI:** 10.3389/fpsyg.2025.1568911

**Published:** 2025-07-09

**Authors:** Alessia Dorigoni, Pier Luigi Giardino

**Affiliations:** ^1^Department of Neuroscience, Imaging and Clinical Sciences, University of Studies G. d'Annunzio Chieti and Pescara, Chieti, Italy; ^2^Department of Economics and Management, University of Trento, Trento, Italy

**Keywords:** Artificial intelligence (AI), human-AI communication, authenticity perception, emotional proximity, AI anthropomorphism

## Abstract

This study investigates how anthropomorphism and source attribution shape perceptions of creativity, authenticity, and moral respect in emotionally significant communication. Drawing on theories of human-machine communication and symbolic value, we examine whether messages are evaluated differently depending on who—or what—is believed to have authored them. Across two experimental studies, we manipulated both the emotional context (childbirth vs. terminal illness) and the attributed message source (close friend, florist, Google, or ChatGPT). Study 1 used a within-subject design to compare message evaluations before and after source disclosure; Study 2 disclosed the source at the outset. Results show that emotional proximity significantly enhances perceived communicative value, while attribution to artificial or emotionally distant sources reduces it. Anthropomorphic cues temporarily elevate AI evaluations but collapse upon disclosure, particularly in high-stakes contexts. Attribution to ChatGPT led to the steepest declines in authenticity and moral respect, underscoring the symbolic and ethical limitations of AI in relationally charged settings. Our findings contribute to the literature on AI-human interaction by theorizing anthropomorphism as a double-edged attributional mechanism and offer practical insights for the deployment of generative AI in domains requiring emotional sensitivity, care, and symbolic coherence.

## 1 Introduction

Artificial Intelligence (AI)—commonly defined as the capacity of machines to imitate intelligent behavior (Choudhury et al., 2020)—has evolved from a tool for scientific and military problem-solving into a ubiquitous presence across both professional and personal domains. From curating social media feeds and optimizing logistics to assisting doctors with diagnoses and generating art, AI increasingly participates in tasks that were once considered exclusively human (Cristofaro and Giardino, 2025). As AI systems grow more capable and widespread, they are not only altering what work gets done, but also reshaping how humans interpret and emotionally engage with the outputs they produce (Dorigoni, 2025).

A growing body of research suggests that emotional responses to AI-generated outputs are not solely a function of technical quality or usefulness. Rather, they are also shaped by how people perceive the process of creation, including who—or what—is behind the output, and how much care, creativity, or emotional investment is presumed to be involved. Recent findings, for instance, indicate that while AI can generate architecturally impressive or emotionally evocative designs, these outputs may be received differently depending on the viewer's expertise and expectations (Zhang et al., 2024). Similarly, AI-generated empathetic messages have been shown to outperform even human-written ones in terms of perceived responsiveness and compassion (Ovsyannikova et al., 2025), yet skepticism remains about their authenticity. These tensions underscore an important and still open question: *what makes AI-generated outputs feel emotionally real*?

Building on this concern, we examine how anthropomorphism operates in emotionally charged settings where human-like qualities are expected, and often demanded, from communicators. Central to this puzzle is the role of anthropomorphism, or the attribution of human-like characteristics such as emotions, intentions, and communicative style to non-human agents. Although anthropomorphism was not directly manipulated in our study, we conceptualize it as an emergent interpretive process, activated when symbolically rich content is unexpectedly attributed to a non-human source. This framework draws on prior research suggesting that anthropomorphism is often not driven by explicit cues, but by contextual and motivational factors that lead individuals to infer human-like qualities such as emotion or intention (Epley et al., 2007). Even minimal cues can be sufficient to trigger such attributions, particularly in emotionally salient or morally charged contexts (Nass and Moon, 2000). In our scenario-based design, we expected that learning an emotionally expressive message had been generated by an AI (rather than a human) would spontaneously elicit these inferences, shaping perceptions of authenticity, respect, and symbolic value. As AI enters domains of emotional significance, such as counseling or personal communication, anthropomorphism may serve as a psychological mechanism that influences how users evaluate the authenticity, trustworthiness, and symbolic meaning of what AI produces. Theoretical work on human-machine communication suggests that AI is no longer merely a tool but a new type of communicative actor—one that requires users to navigate unfamiliar social and moral boundaries (Guzman and Lewis, 2020; Sundar and Lee, 2022). Yet we still know little about when and how these human-like features actually shape emotional responses to AI outputs, particularly in contexts that demand care, respect, and emotional resonance.

To investigate how anthropomorphism influences emotional responses to AI-generated outputs, we conducted two complementary experiments examining individuals' evaluations of emotionally significant messages. These messages varied systematically by source (i.e., self, friend, stranger, AI) and medium (i.e., typed vs. handwritten), allowing us to assess how perceptions of creativity, respectfulness, and authenticity are shaped by who is believed to have authored the message and how it was delivered. Following prior literature, we define our key constructs as follows: Creativity is defined as “the ability to generate novel and appropriate ideas, processes, or solutions,” with an emphasis on its function within social interactions and symbolic communication (Bartels and Dubina, 2020). Authenticity refers to that which is perceived as “real,” “genuine,” or “true,” shaping how messages are interpreted in emotionally significant contexts (Newman and Smith, 2016). To respect a person, at least from a Kantian perspective, means recognizing their intrinsic, indeed, priceless, worth or dignity, and treating them as ends in themselves rather than merely as means (Heubel and Biller-Andorno, 2005). While participants assessed these traits via message-level features (e.g., text content), they implicitly inferred them as characteristics of the sender, particularly after learning the source identity (human, AI, or stranger). We embedded this design within two emotionally charged fictitious scenarios: (i) the birth of a child and (ii) the diagnosis of a terminal illness. These contexts were chosen because they naturally heighten expectations of care, effort, and symbolic meaning, making them well-suited for isolating the emotional effects of anthropomorphism in AI-generated outputs. In such settings, recipients tend to infer not only meaning from the content itself, but also emotional investment from the act of message creation—offering a powerful lens through which to examine how users respond when AI steps into roles traditionally reserved for human empathy. In doing so, we make three contributions. First, we advance theory on anthropomorphism in AI by demonstrating how perceptions of human-likeness interact with emotional and relational cues to shape user responses. Second, we enrich the literature on symbolic value and perceived effort in communication, revealing how these elements influence emotional engagement with AI. Third, we offer practical insights for the design of AI systems intended to operate in emotionally sensitive settings, where trust and empathy are critical.

## 2 Theoretical background and hypotheses development

### 2.1 AI and the perception of creativity

Creativity is a multifaceted construct in human communication, typically defined as the ability to produce outputs that are novel, valuable, and contextually appropriate (Runco and Jaeger, 2012). Beyond its technical dimensions, creativity encompasses relational, symbolic, and affective meanings. In emotionally charged interpersonal contexts creative expression signifies not only originality but also attunement, emotional engagement, and relational presence. Messages in these situations are evaluated not just for their aesthetic quality but for their capacity to reflect shared meaning, symbolic depth, and intentional emotional investment (Boden, 2004).

AI systems, particularly large language models and generative AI, have demonstrated an increasing capacity to produce outputs that meet the surface criteria for creativity. Through extensive training on large datasets and probabilistic modeling, these systems can compose poems, generate narratives, and simulate symbolic expression with stylistic fluency and contextual relevance (McCormack et al., 2019). High-profile cases, such as the introduction of “Y3000,” a futuristic beverage co-created by Coca-Cola and AI, exemplify AI's capacity to enter domains once thought to be exclusively human. In specific task-oriented settings, AI-generated content is often accepted, if not admired, for its ingenuity, efficiency, and technical creativity.

However, empirical evidence suggests that AI's perceived creativity remains highly contingent on attribution. In controlled experiments, audiences routinely devalue creative work when informed it was generated by AI rather than a human—even when the output is identical (Zhang et al., 2024). This discrepancy is magnified in emotionally salient contexts, where creativity is associated with human subjectivity, intentionality, and existential insight. The absence of an experiential, feeling subject behind the message renders AI-generated creativity emotionally hollow or symbolically dissonant in the eyes of many recipients (Ovsyannikova et al., 2025).

Anthropomorphism offers a theoretical lens to explain these discrepancies. When AI systems exhibit human-like qualities (e.g., using emotionally expressive language, demonstrating contextual sensitivity, or appearing embodied in avatars) users are more inclined to interpret their outputs as stemming from intentional, affectively invested agents (Epley et al., 2007). This enhances the perceived legitimacy of the AI's creative capacity. Conversely, when anthropomorphic cues are absent or weak, AI is interpreted as a mere algorithmic tool, capable of generating novel content but devoid of the symbolic, social, and emotional grounding that authenticates creativity in human communication (Fussell et al., 2008). In this light, the perception of AI creativity is not determined by the intrinsic quality of the output but by the degree to which the system is seen as a quasi-human creator.

### 2.2 AI and the attribution of authenticity

Authenticity is commonly conceptualized as the alignment between one's internal state and external expression (Erickson, 1995). It fosters trust, emotional connection, and social bonding, and is particularly salient in affect-laden contexts where individuals seek reassurance, empathy, or moral solidarity (Kreber et al., 2007). In situations involving health crises or major life events, authenticity is expected not merely as a stylistic feature of communication but as a relational and ethical imperative—a demonstration of emotional sincerity and interpersonal commitment (Harter, 2002).

Advances in AI have enabled systems to simulate authenticity with increasing fluency. Through affective computing and natural language generation, AI can mimic emotional tone, simulate empathy, and personalize messages based on user history or situational context (Picard, 1997). Design elements such as emotionally expressive avatars, human-like voices, and adaptive dialogue systems can increase an AI's social presence, making interactions feel more natural and emotionally resonant (Bickmore et al., 2009). These anthropomorphic features exploit humans' innate tendency to engage socially even with non-human agents (Reeves and Nass, 1996), thereby fostering perceived authenticity in AI-human communication (Gambino et al., 2020).

However, perceptions of AI authenticity are highly unstable and can quickly collapse under certain conditions. When users discover that a seemingly heartfelt message was generated by an AI, they often retrospectively reappraise it as insincere, manipulative, or emotionally vacuous (Logg et al., 2019). This is especially pronounced in contexts where the expression of authenticity is symbolically and relationally significant. In such cases, authenticity is inferred not merely from emotional tone but from the perceived identity and relational investment of the sender (Van Boven et al., 2003).

Anthropomorphism serves as a double-edged mechanism in shaping perceptions of authenticity. On one hand, anthropomorphic features increase the plausibility of social intentionality, encouraging users to attribute sincerity and emotional presence to the AI (Waytz et al., 2010). On the other hand, when these features are incongruent with users' expectations or are discovered to be artificially constructed, they can evoke perceptions of inauthenticity or ethical manipulation (Złotowski et al., 2017). The more human-like the AI appears, the higher the standards it is held to—and the greater the disappointment if it fails to meet those standards. This dynamic mirrors the “authenticity valley”, phenomenon that is conceptually inspired by, but distinct from, the uncanny valley (Mori, 1970; Mori et al., 2012; MacDorman and Chattopadhyay, 2016), in which AI systems that appear almost, but not quite, authentically human evoke discomfort, distrust, and skepticism. The “authenticity valley” describes a non-linear dip in perceived authenticity and emotional resonance that occurs when a message, initially interpreted as meaningful or respectful, is later revealed to originate from a non-human or symbolically distant source (e.g., an AI system or impersonal institution). While the uncanny valley is rooted in perceptual-motor mismatches and threat-detection systems (e.g., Moosa and Ud-Dean, 2010), the authenticity valley emerges from violations of relational expectations and symbolic incongruity in emotionally salient contexts. The implications are profound: in emotionally significant communication, the symbolic coherence between form, content, and perceived identity is essential. Anthropomorphism can enhance this coherence but also destabilize it when overextended or underdelivered (Nass and Moon, 2000). Psychologically, the authenticity valley may be driven by expectancy violation theory (Burgoon, 1993) and the breakdown of moral projection: when individuals attribute human-like intentionality or empathy to a message, discovering it comes from a non-human agent (e.g., an AI) disrupts this projection, leading to a sharp reevaluation of its emotional and moral value. It may also involve reactance or betrayal aversion (Bohnet and Zeckhauser, 2004), especially when the context involves vulnerability or moral significance. The authenticity valley may operate across multiple modalities. For instance, in the auditory domain, Schroeder and Epley (2016) showed that adding a human voice to a machine-generated script increased the perception of a humanlike mind behind the message, suggesting that voice can enhance or disrupt perceived authenticity. Similarly, Mitchell et al. (2011) demonstrated that a mismatch between humanlike faces and synthetic voices induces discomfort, highlighting that incongruence across modalities can produce a multimodal version of what we called authenticity valley. These findings may imply that when language, tone, or even olfactory cues (e.g., artificial scents simulating emotional contexts) fail to align with expectations of human authenticity, they may trigger a dip in perceived sincerity, emotional resonance, or moral respect.

### 2.3 AI and the perception of moral respect

Respect is a foundational concept in moral psychology and organizational behavior, denoting recognition of the other's dignity, autonomy, and emotional significance (Honneth, 1995; Tyler and Blader, 2003). In interpersonal communication, respect is not only conveyed through language and tone but also through attentiveness to context, appropriateness of emotional expression, and acknowledgment of the other's subjectivity (Dillon, 1992). In emotionally salient settings respectful communication is understood as a moral obligation, not simply a social courtesy. Such situations demand that communicative acts are sensitive to symbolic meaning and the relational depth of the moment.

AI systems are increasingly deployed in domains where respectful communication is critical. Virtual agents are now used in caregiving, healthcare, and counseling contexts, settings in which users expect moral sensitivity and emotional support (De Vault et al., 2014; Lucas et al., 2014). In certain cases, AI can be perceived as more respectful than human agents due to its consistency, impartiality, and lack of judgment. For example, individuals often disclose more sensitive information to AI-driven therapists or health chatbots than to human counterparts, citing a sense of psychological safety and reduced fear of stigma (Miner et al., 2016). When AI systems are designed with anthropomorphic features (e.g., human-like voices, emotionally expressive facial animations, or responsive conversational cues) users report higher perceived attentiveness, empathy, and moral engagement (Gambino et al., 2020; Nass and Moon, 2000).

However, the simulation of respect by AI is contingent on user expectations and contextual salience. In symbolically rich or morally charged interactions, users often expect the message sender to possess not only emotional intelligence but also moral awareness. In this regard, when an AI-generated message of sympathy, encouragement, or congratulations is later revealed to have originated from a non-human source, recipients may experience disappointment, discomfort, or even moral unease, especially if the AI exhibited highly anthropomorphic features (Luo et al., 2019; Bigman and Gray, 2018). The attribution of moral respect depends not only on the linguistic form of the message but on the perceived moral agency of the sender. An artificial source, however convincingly human-like, may be perceived as illegitimate or inadequate in fulfilling the moral role expected in such contexts.

Anthropomorphism serves as a key explanatory lens for these divergent reactions. When users perceive AI systems as intentional agents with human-like emotions and awareness, they may project onto them moral expectations typically reserved for human beings (Waytz et al., 2014). Anthropomorphic design encourages users to infer empathy, compassion, or care—social cues that make the AI appear morally engaged. However, the more human-like the AI becomes, the higher the moral standards it is held to, and the greater the disappointment if it fails to deliver appropriately respectful communication (Złotowski et al., 2017; Seeger et al., 2021). This dynamic is particularly precarious in high-emotion or symbolically loaded scenarios, where moral breaches are not easily dismissed. As with authenticity, respect is not assessed solely on the content of a message but through coherence between content, context, and the moral legitimacy of the source. Anthropomorphism can enhance this coherence by creating the illusion of moral agency, but it also raises the stakes: when AI fails to meet the expectations it sets through its human-like design, users may experience a profound sense of moral incongruity.

### 2.4 Hypotheses development

Building on the preceding theoretical sections, we propose that perceptions of creativity, authenticity, and moral respect in emotionally significant communication are not reducible to assessments of linguistic quality or stylistic sophistication. Rather, they are shaped by attributional processes through which message recipients infer the sender's intentionality, emotional investment, and relational identity (Van Boven et al., 2003; Harter, 2002). In affect-laden interpersonal contexts, individuals evaluate not only *what* is said but also *who* is imagined to be saying it. Messages believed to originate from emotionally close others—such as a close friend—are more likely to be perceived as creative, authentic, and respectful. These perceptions emerge not from intrinsic textual features, but from symbolic co-construction between message content and the inferred identity of the sender (Logg et al., 2019; Ovsyannikova et al., 2025). Conversely, when the source is perceived as emotionally distant or non-human, evaluations often shift toward more instrumental or impersonal interpretations, resulting in symbolic and emotional devaluation.

**Hypothesis 1:** *The perceived source of a message will significantly influence individuals' judgments of creativity, authenticity, and moral respect*.

This attributional asymmetry becomes particularly salient in two-stage evaluations, in which initial impressions are formed without source knowledge and later reappraised upon source disclosure. Empirical research indicates that individuals often assume emotionally expressive or well-crafted messages stem from relationally close senders (Zhang et al., 2024). This implicit assumption anchors expectations and creates a relational baseline against which subsequent attributional information is judged. When the actual sender is revealed to be a distant actor (e.g., a service provider or anonymous website) or a non-human agent (e.g., a generative system), recipients may experience a violation of these expectations, prompting downward reassessments. These effects are especially pronounced for authenticity and respect, which hinge on perceptions of emotional sincerity and moral engagement (Erickson, 1995; Kreber et al., 2007). The discovery that a seemingly heartfelt message originates from an artificial source can render the message emotionally hollow or symbolically dissonant (Ovsyannikova et al., 2025). Even when the sender is a human actor without relational proximity, such as a third-party intermediary, the lack of emotional connection may dilute the message's perceived social meaning. By contrast, confirmation that a message came from a close relational source may stabilize or even enhance perceptions of authenticity and respect. Perceptions of creativity, in contrast, may be more resilient to attributional change, given that originality may be judged more on output features than on relational meaning (Boden, 2004).

**Hypothesis 1a:** *Evaluations of creativity, authenticity, and moral respect are expected to decline when the message source is revealed to be emotionally distant or non-human, but remain stable or improve when the source is relationally close and emotionally invested*.

Attribution also shapes the *magnitude* of evaluative change. This dynamic reflects not merely whether judgments are revised, but how much they are revised—a process that reveals how deeply attributional cues affect interpretive coherence. Among the strongest disruptions are those triggered by artificial sources. Because these systems lack experiential subjectivity, emotional intentionality, and symbolic legitimacy in morally or emotionally significant contexts, they often produce the greatest evaluative dissonance upon disclosure (Złotowski et al., 2017; Bigman and Gray, 2018). Messages from stranger but human sources may lead to more moderate reappraisal, given the ambiguity surrounding relational and emotional intent. In contrast, confirmation that a message was authored by a close friend is expected to reinforce coherence between content and sender, minimizing dissonance and possibly enhancing perceived authenticity or moral presence.

**Hypothesis 1b:** *The magnitude of evaluative change following source disclosure is expected to vary by source type, with the steepest declines occurring when the sender lacks human or emotional presence, and minimal or positive shifts when the sender is relationally close*.

These attributional effects are further modulated by anthropomorphic cues. When non-human systems are designed to mimic human qualities—through expressive language, contextual sensitivity, or embodied avatars—they increase perceived intentionality and emotional engagement (Epley et al., 2007; Waytz et al., 2010). These cues can elevate perceptions of creativity, authenticity, and moral respect in the short term, especially when the source remains undisclosed. However, the same cues can backfire once the sender is revealed to be artificial. The initial plausibility of emotional or moral investment collapses, producing feelings of manipulation, symbolic incoherence, or even ethical discomfort (Mori et al., 2012; Złotowski et al., 2017). This pattern aligns with the concept of an “authenticity valley”, a parallel to the uncanny valley in which near-human expressiveness fails not because of output quality, but because of dissonance between form and source identity (Nass and Moon, 2000). The more human-like the system appears, the more it invites normative expectations—and the more pronounced the disappointment when those expectations are unmet. In comparative evaluations across multiple message sources, these attributional and anthropomorphic dynamics give rise to a perceived hierarchy of communicative legitimacy. Messages believed to originate from a close friend are expected to score highest across the three constructs, as they align most closely with norms of relationally invested expression. Messages from a stranger human sources, such as service personnel or anonymous online users, may be seen as socially functional but emotionally neutral. By contrast, messages from artificial or impersonal sources—such as a web page or an AI system—are expected to rank lowest, reflecting their symbolic detachment from emotional and moral presence.

**Hypothesis 2:** *When comparing message sources, individuals will prioritize those perceived as emotionally close, followed by emotionally neutral human sources, with non-human or impersonal sources evaluated lowest on creativity, authenticity, and moral respect*.

## 3 Methodology

To explore how people perceive the authenticity, creativity, and respectfulness of emotionally significant messages based on their source and delivery medium, we conducted two complementary experimental studies using vignette-based designs administered online via Prolific. This methodological approach aligns with a long-standing tradition in behavioral and moral psychology that uses emotionally salient hypothetical scenarios to investigate judgment and decision-making. Classic examples include the “Asian Disease Problem” (Tversky and Kahneman, 1981) and the “Trolley Problem” (e.g., Foot, 1967), which similarly place participants in emotionally charged yet hypothetical dilemmas. In addition to classic dilemmas in moral psychology, our methodological approach aligns with more recent vignette-based studies exploring emotionally salient judgments in applied settings. For example, Lawton et al. (2010) used hypothetical scenarios describing antenatal care outcomes and provider-patient relationships to examine participants' judgments of responsibility, blame, and likelihood of filing a complaint. Their findings demonstrate that individuals can meaningfully evaluate complex moral and relational constructs using vignette-based materials, even in emotionally charged healthcare contexts. A substantial body of literature employs vignette-based methodologies to investigate medical decision-making, professional judgement, and social evaluations across a variety of contexts. Vignettes allow researchers to systematically manipulate key variables in hypothetical scenarios, enabling controlled investigation of complex processes such as attribution, bias, and ethical decision-making. For example, Bachmann et al. (2008) conducted a systematic review of vignette studies examining medical choices and caregiver behavior, highlighting their utility in assessing clinical decision-making processes. Taylor (2006) discussed the factorial survey method as a robust approach to studying professional judgment using vignettes. Similarly, Madsen et al. (2016) used factorial vignettes to examine whether therapeutic judgment is influenced by a patient's socioeconomic status. Other studies have used vignette methods to assess competence to consent (Vellinga et al., 2004), explore cultural bias in diagnosis (Mikton and Grounds, 2007). These examples confirm that vignettes are an effective, ethically sound and widely validated tool for studying how professionals and laypeople make complex judgments in controlled yet realistic contexts. Following best practices in experimental vignette methodology (Aguinis and Bradley, 2014), participants were randomly assigned to conditions that manipulated the emotional context (e.g., childbirth vs. terminal illness) and the message source (e.g., friend, stranger, florist, Google, AI). In both studies, participants evaluated emotionally meaningful notes addressed to a woman named Anna, hospitalized under varying circumstances. The experimental designs allowed us to capture not only initial perceptions but also how those perceptions shifted upon learning who had authored the message—shedding light on how source attribution shapes perceived emotional value. Participants rated each message on creativity, authenticity, and respect using Likert-type scales, and emotional responses were measured using the PANAS scale. Demographic data and participants' attitudes toward artificial intelligence were also collected. Study 1 used a within-subject design, asking participants to rate the same message before and after source disclosure across six experimental conditions. Study 2 employed a between-subjects design to compare judgments across four different message sources, enabling comparative evaluations of how varying origins influence emotional reception.

### 3.1 Study 1

The study involved a total of 120 participants recruited via the Prolific platform. Participants were evenly divided into six experimental conditions, with 20 participants per condition. The experimental design followed a 2 × 3 factorial structure: two hospitalization scenarios (i.e., serious illness and childbirth) and three message sources (AI, Written by Roberta, Florist).

Participants were initially presented with a scenario describing a woman, Anna, in the hospital. The scenario had two variations: (i) Anna is hospitalized after giving birth to her first child, (ii) Anna is hospitalized due to being terminally ill and receiving palliative care.

After reading the scenario, participants were shown a printed note that Anna had received from her friend Roberta, along with a bouquet of flowers. The note read: “*Dear Anna, I'm sending you a warm thought during this time. I hope you can find strength and love in the warmth of those who care for you. I'm with you with all my heart. With love, Roberta.”*

Participants were then asked to evaluate the note on a 5-point Likert scale in terms of creativity, authenticity, and respect. Subsequently, participants were informed that the note was written under one of the following three conditions:

a. Roberta wrote the note herself.
b. Roberta used ChatGPT (AI) to write the note.
c. Roberta asked a stranger, the florist, to write the note.

After learning the source of the note, participants re-evaluated the message in terms of creativity, authenticity, and respect based on the new information about its origin. They were also asked to report positive and negative emotions using the Italian adaptation of the Positive and Negative Affect Schedule (PANAS) developed by Terraciano et al. (2003), based on the original scale by Watson et al. (1988).

Additionally, participants provided information about their personal connection to artificial intelligence and demographic data.

The sample included 72 males, 45 females, and 3 participants who preferred not to disclose their gender. The mean age of participants was 34.37 years, with an age range of 21 to 66 years. Initially, the dataset included a larger number of participants, but those who completed the questionnaire in an excessively short time were excluded to ensure response quality.

### 3.2 Study 2

The study involved a total of 40 participants recruited via the Prolific platform. Participants were evenly divided into two experimental conditions.

Participants were initially presented with a scenario describing a woman, Anna, in the hospital. The scenario had two variations: (i) Anna is hospitalized after giving birth to her first child, and (ii) Anna is hospitalized due to being terminally ill and receiving palliative care. After reading the scenario, participants were shown a printed note that Anna had received from her friend Roberta, along with a bouquet of flowers. The note read: “*Dear Anna, I'm sending you a warm thought during this time. I hope you can find strength and love in the warmth of those who care for you. I'm with you with all my heart. With love, Roberta.”*

Finally, participants were asked to rank messages written by the best friend, the florist, Google, and artificial intelligence in terms of creativity, authenticity, and respect.

## 4 Results

### 4.1 Study 1

Study 1 investigated how perceived authorship shapes evaluations of emotionally significant messages, focusing on three constructs: creativity, authenticity, and moral respect. Participants were randomly assigned to one of six conditions in a 2 (emotional context: childbirth vs. terminal illness) × 3 (source: friend, a stranger person, artificial intelligence) factorial design. Each participant initially evaluated an identical message—presented as if written by a close friend—before learning the actual source: either a close friend (Roberta), a stranger (a florist), or an AI tool. Participants then re-evaluated the same message, allowing for within-subject comparisons and subsequent analysis of attributional effects.

#### 4.1.1 Perceived creativity

**Figure 1:**
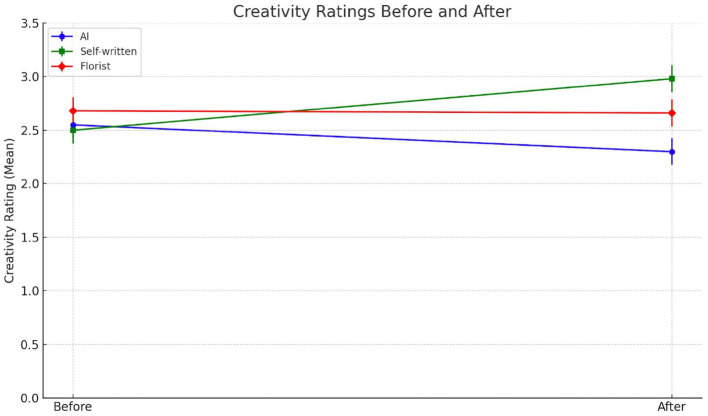
Creativity ratings before and after the source was revealed. Participants evaluated the creativity of a supportive message in three conditions: AI-generated (blue), self-written by a friend (green), and written by a florist (red). The dots represent mean creativity ratings before and after revealing the message source.

Creativity, particularly in emotionally charged contexts, is not merely a function of syntactic novelty or linguistic elegance but is tied to perceptions of emotional effort, symbolic intent, and relational meaning. We hypothesized that source attribution would shape these perceptions, with authorship by a close friend enhancing the perceived creativity of the message, and attribution to a socially distant or non-human source reducing it. In the friend condition, the discovery that the message was authored by Roberta significantly elevated creativity ratings—from a pre-disclosure mean of 2.50 (SD = 0.91) to 2.98 (SD = 1.21), *t*_(39)_ = −3.68, *p* < 0.001 (Figure 1). This upward shift suggests that creativity in this context is co-constructed with relational inference: participants interpreted the same text as more creative once they believed it was produced by someone emotionally close, likely attributing greater symbolic intentionality and expressive investment. By contrast, in the AI condition, creativity ratings declined modestly following source disclosure, from 2.55 (SD = 0.96) to 2.30 (SD = 1.22), *t*_(39)_ = 1.50, *p* = 0.14. Although not statistically significant, the direction of the shift aligns with prior findings that creative outputs, when attributed to non-human systems, are perceived as technically proficient but emotionally shallow. Participants may have acknowledged the surface-level fluency of the message while discounting its expressive depth. The unknown person's condition showed no significant change in perceived creativity (pre-disclosure: 2.68, SD = 1.00; post-disclosure: 2.65, SD = 1.17), *t*_(39)_ = 0.21, *p* = 0.84. This stability suggests that while the florist was recognized as human, their lack of emotional proximity rendered the message creatively neutral—neither enhanced by personal relevance nor diminished by artificial detachment (due to the lack of emotional connection for its non-human origin). To ensure conceptual clarity, we adopt a consistent terminology throughout the paper. Specifically, we focus on two core constructs: emotional proximity and relational closeness. Emotional proximity refers to the perceived emotional resonance, warmth, or attunement conveyed through a message, regardless of the sender's identity. Relational closeness refers to the perceived familiarity, intimacy, or personal connection between the message sender and the recipient. These two constructs capture complementary aspects of perceived authenticity in emotionally significant contexts.

These results support Hypothesis 1a in the domain of creativity: only attribution to a close friend produced a meaningful shift in perception. Attributions to AI and socially distant humans failed to reframe the message as more (or less) creative, reinforcing the notion that in emotionally meaningful exchanges, creativity is evaluated not just on the basis of linguistic markers but through a lens of inferred emotional investment.

#### 4.1.2 Perceived authenticity

**Figure 2:**
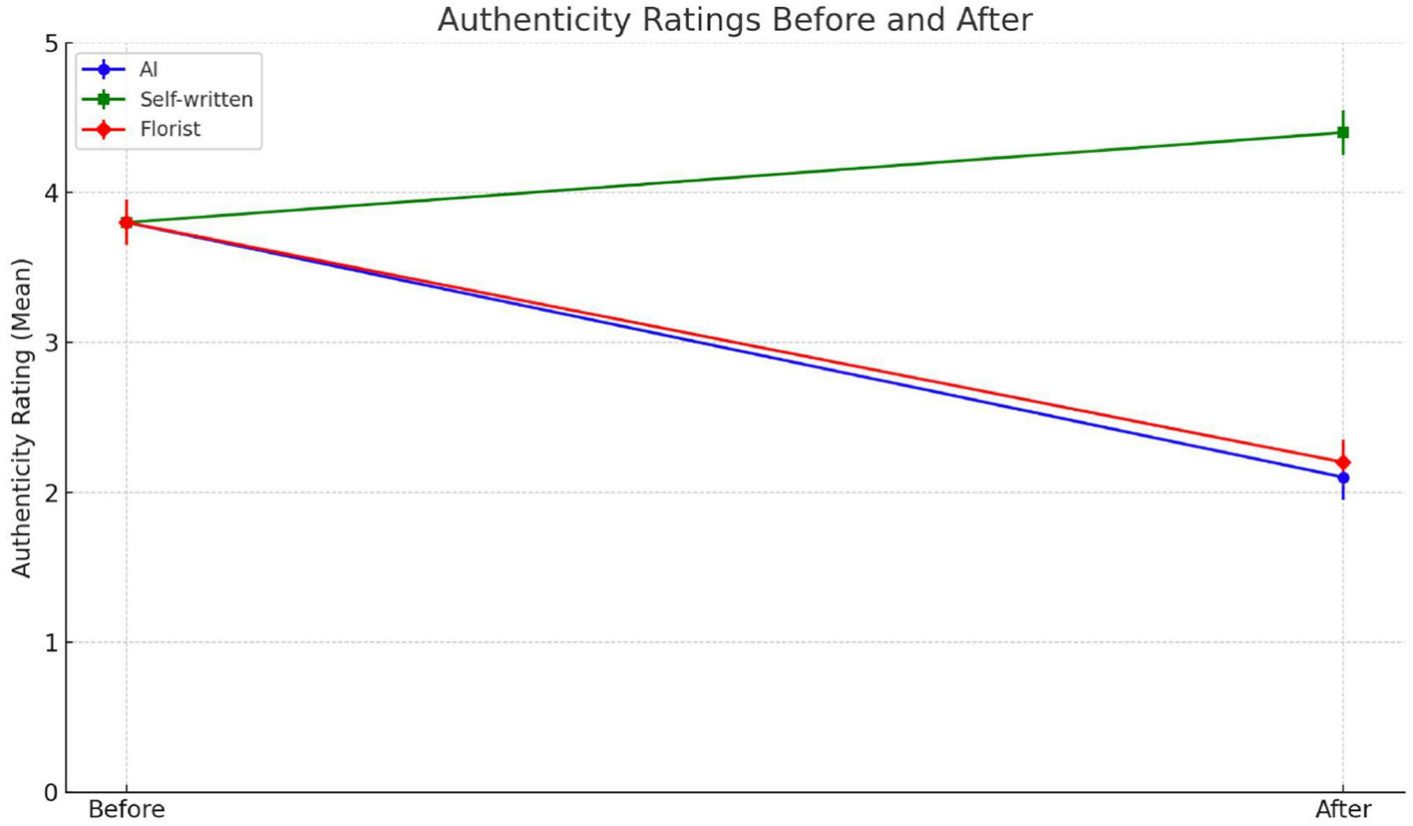
Authenticity ratings before and after the source was revealed. Participants evaluated the authenticity of a supportive message in three conditions: AI-generated (blue), self-written by a friend (green), and written by a florist (red). The dots represent mean authenticity ratings before and after revealing the message source.

Authenticity was the dimension most sensitive to source attribution. Rooted in the alignment between internal sincerity and external expression, authenticity in relational communication signals emotional presence and symbolic commitment. We expected that attributions to emotionally close individuals would enhance perceived authenticity, while non-human or socially distant sources would undermine it. In the AI condition, authenticity ratings fell sharply after disclosure—from 3.78 (SD = 1.07) to 2.08 (SD = 1.33), t_(39)_ = 7.78, *p* < 0.001. This striking decrease reflects the dissonance between a message initially perceived as sincere and its later reclassification as algorithmically generated. The attributional shift appears to disrupt the assumption of emotional intentionality, leading participants to reinterpret the message as affectively hollow despite unchanged content (Figure 2). In contrast, in the friend condition, participants' authenticity ratings increased significantly—from 3.83 (SD = 0.93) to 4.35 (SD = 0.74), t_(39)_ = −5.19, *p* < 0.001. This suggests that once the authorship was confirmed to stem from an emotionally close sender, the message gained symbolic credibility and affective resonance. The perceived sincerity of the message was reinforced by congruence between content and source identity. The unknown person condition produced results similar to the AI condition: authenticity dropped from 3.80 (SD = 1.14) to 2.15 (SD = 1.27), t_(39)_ = 7.42, *p* < 0.001. Though less severe than in the AI case, the decline reveals that social distance—regardless of human status—erodes the perceived genuineness of emotionally supportive messages. Taken together, these findings underscore the relational and inferential nature of authenticity. Attribution to a close friend stabilized and enhanced perceived sincerity; attribution to AI or an unknown human undermined it. These results offer strong support for Hypothesis 1a in the domain of authenticity, reinforcing that judgments of sincerity are inseparable from the perceived emotional proximity and moral presence of the source.

#### 4.1.3 Perceived moral respect

**Figure 3:**
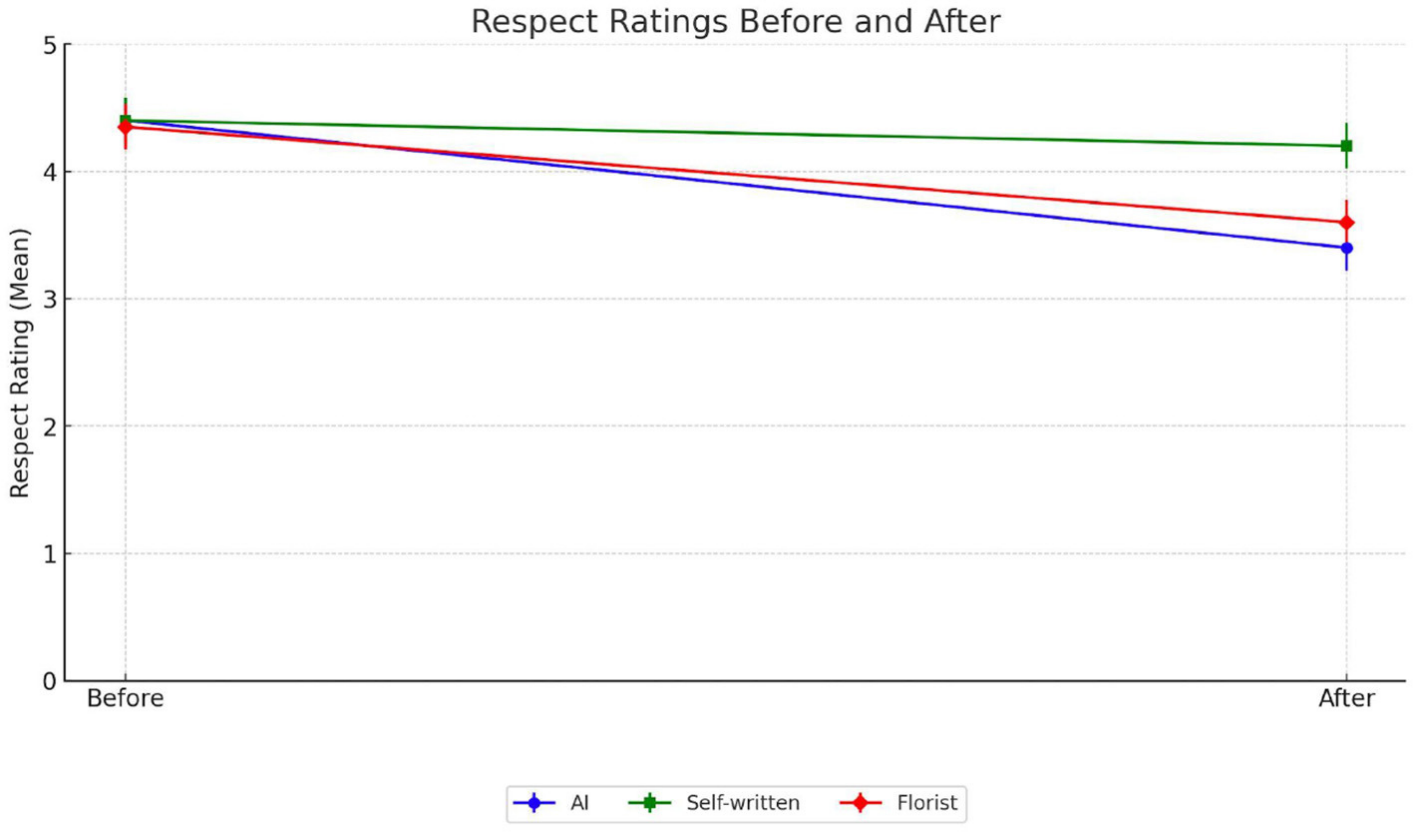
Respect ratings before and after the source was revealed. Participants evaluated the respect of a supportive message in three conditions: AI-generated (blue), self-written by a friend (green), and written by a florist (red). The dots represent mean respect ratings before and after revealing the message source.

Respect, as a moral and symbolic gesture, conveys recognition of the other's emotional gravity, especially in contexts like childbirth or terminal illness. Unlike creativity or authenticity, respect is often associated with social norms of attentiveness and appropriateness. We hypothesized that respect, too, would be shaped by source attribution, albeit potentially in a more normatively anchored way. In the AI condition, respect ratings dropped from 4.42 (SD = 0.90) to 3.40 (SD = 1.53), t_(39)_ = 4.95, *p* < 0.001. The message, initially deemed respectful based on tone and content, lost symbolic legitimacy once its origin was revealed to lack moral agency (Figure 3). Participants appear to infer that respectful gestures require not just formal politeness but a capacity for moral awareness—something AI, by its very nature, cannot convincingly provide. Interestingly, in the friend condition, the attribution of authorship to Roberta led to a small but significant decline in respect ratings, from 4.42 (SD = 0.84) to 4.22 (SD = 1.00), t_(39)_ = 3.12, *p* < 0.003. This deviation from the pattern observed for creativity and authenticity may reflect a recalibration effect. Participants may have initially assumed the message was authored by a close friend, thus already anchoring it at a high level of respect. When this assumption was confirmed, participants may have reassessed the message more critically—perhaps finding it somewhat conventional or lacking in expressive depth. In the unknown person condition, respect ratings also decreased—from 4.35 (SD = 1.05) to 3.60 (SD = 1.19), t_(39)_ = 4.21, *p* < 0.001. Though still human, the florist's lack of relational connection reduced the moral and symbolic weight of the gesture. These findings suggest that respect is not derived from language alone, but from a perceived alignment between the gravity of the situation and the legitimacy of the sender. Thus, while respect was initially high across all conditions, only the attribution to a friend preserved its symbolic legitimacy. Both AI and unknown human authorship led to a loss of perceived moral presence, reinforcing Hypothesis 1A in this domain.

It is important to note that respect scores decreased in all three conditions after the source was revealed. However, the decline in the friend condition, although statistically significant, was noticeably smaller in magnitude than the drops observed in the AI and unknown person conditions. In terms of authenticity, both artificial intelligence and the florist differ significantly from the condition where the friend, Roberta, wrote the card herself, demonstrating that Roberta's personal involvement in writing the card results in a significantly higher perception of authenticity and respect. A deeper analysis based on participants' connection to artificial intelligence could not be performed, as the majority of responses (81) to the statement “*I feel personally connected to AI software*” indicated disagreement or strong disagreement. Including neutral responses brings this number to 109 participants. The distribution was therefore highly skewed, as no participants reported a strong connection to artificial intelligence.

Furthermore, while Hypothesis 1a focused on within-subject shifts in evaluation following source disclosure, Hypothesis 1b aimed to assess whether the magnitude of these shifts varied systematically across different source conditions. The underlying proposition is that the symbolic coherence—or lack thereof –between message content and perceived sender identity elicits not only directional changes in judgment but also differences in the *intensity* of evaluative recalibration. To test this, we computed absolute delta scores (i.e., the absolute value of the difference between pre- and post-disclosure ratings) for each participant across the three focal dimensions: creativity, authenticity, and moral respect. These delta scores serve as a proxy for attributional disruption—capturing the extent to which reattributing authorship reshapes the interpretive weight of the message. One-way ANOVAs were conducted on these delta scores across the three source conditions (friend, unknown person, AI), followed by Tukey's HSD *post hoc* tests to determine the locus of significant differences.

#### 4.1.4 Perceived creativity

For creativity, the ANOVA revealed a significant effect of message source on delta scores, *F*_(2, 114)_ = 3.20, *p* = 0.045. *Post hoc* comparisons indicated that the shift in creativity evaluations was significantly greater in the close friend condition than in either the AI or unknown person conditions (*p* < 0.05 for both), which did not differ significantly from each other. This finding suggests that only when authorship was attributed to an emotionally close source did participants substantially reappraise the creative value of the message. By contrast, AI and unknown person sources failed to provoke meaningful reinterpretation, likely due to the absence of perceived emotional investment or relational symbolism. These results underscore that perceptions of creativity in emotionally salient contexts are not solely driven by output quality, but by the attribution of expressive intention—an attribution that is amplified in relationally anchored exchanges.

#### 4.1.5 Perceived authenticity

The largest attributional effects were observed for authenticity. The ANOVA yielded a highly significant result, *F*_(2, 114)_ = 50.66, *p* < 0.001, indicating that the magnitude of change in authenticity perceptions varied substantially by source. Tukey *post hoc* tests revealed that both the AI and unknown person conditions produced significantly larger shifts in authenticity ratings than the close friend condition (*p* < 0.001 and *p* < 0.05, respectively). However, the AI and unknown person groups did not differ significantly from each other. These results reinforce the central theoretical claim that authenticity is particularly sensitive to the perceived emotional intentionality and relational proximity of the sender. Attribution to a non-human or socially distant source disrupted the interpretive coherence of the message, undermining its symbolic congruence and diminishing its perceived sincerity. In contrast, attribution to a close friend stabilized or even enhanced authenticity judgments, reflecting the moral and emotional salience of relational identity in evaluations of communicative authenticity.

#### 4.1.6 Perceived moral respect

A similar pattern emerged for moral respect. The ANOVA revealed a significant main effect of source on respect-related delta scores, *F*_(2, 114)_ = 4.61, *p* = 0.012. *Post hoc* analyses showed that both the AI and unknown person conditions led to significantly greater changes in respect ratings compared to the close friend condition (*p* < 0.05 for both). Again, no significant difference was found between the AI and unknown person conditions. These findings suggest that moral respect—like authenticity—is contingent not just on the content or tone of the message, but on the perceived moral agency and symbolic legitimacy of the sender. Both non-human and socially distant human sources were perceived as lacking the relational authority or moral presence to communicate appropriately in emotionally charged contexts. The close friend condition, by contrast, preserved the symbolic coherence necessary for the message to be interpreted as morally respectful.

Taken together, the results of Hypothesis 1b offer robust empirical support for the central theoretical claim that attributional identity significantly moderates the interpretive weight of emotionally significant communication. Attribution to a relationally close human source buffered or amplified message meaning across all three dimensions. In contrast, attribution to either an AI or a socially distant human undermined perceptions of creativity, authenticity, and respect—particularly in ways that reflect a breakdown in symbolic alignment between sender, message, and context. These patterns confirm that communicative evaluations are deeply relational and attribution-sensitive, and that symbolic misalignment—whether due to artificiality or social anonymity—can fundamentally recalibrate how emotional and moral content is perceived, even when the message itself remains unchanged.

### 4.2 Study 2

Study 2 was not pre-planned but developed in response to the findings of Study 1, with the goal of further disentangling whether participants' reactions to the message stemmed from the impersonal or “mechanical” nature of an AI system, or more broadly from any non-close source. We therefore introduced “Google” as an additional condition to distinguish reactions to an impersonal but human-associated entity from those to a fully artificial one. Given its *post hoc* and exploratory status, Study 2 was designed as a preliminary, hypothesis-generating investigation rather than a confirmatory test. Study 2 builds upon the findings of Study 1 by shifting the analytical lens from within-subject change to between-group comparison. Rather than assessing how evaluations shift after the source of a message is disclosed, Study 2 examines whether the same message is interpreted differently depending on the initially declared source. This design isolates the symbolic and emotional effects of attribution itself, allowing for the identification of source-based biases in how creativity, authenticity, and moral respect are perceived. Participants were randomly assigned to one of four conditions, each attributing the identical message to a different source: a close friend, an unknown person (a florist), an impersonal institutional entity (Google), or a non-human agent (AI system). By holding the content constant, we examine how relational proximity and human vs. non-human status shape interpretive meaning in emotionally significant communication.

#### 4.2.1 Perceived creativity

**Figure 4:**
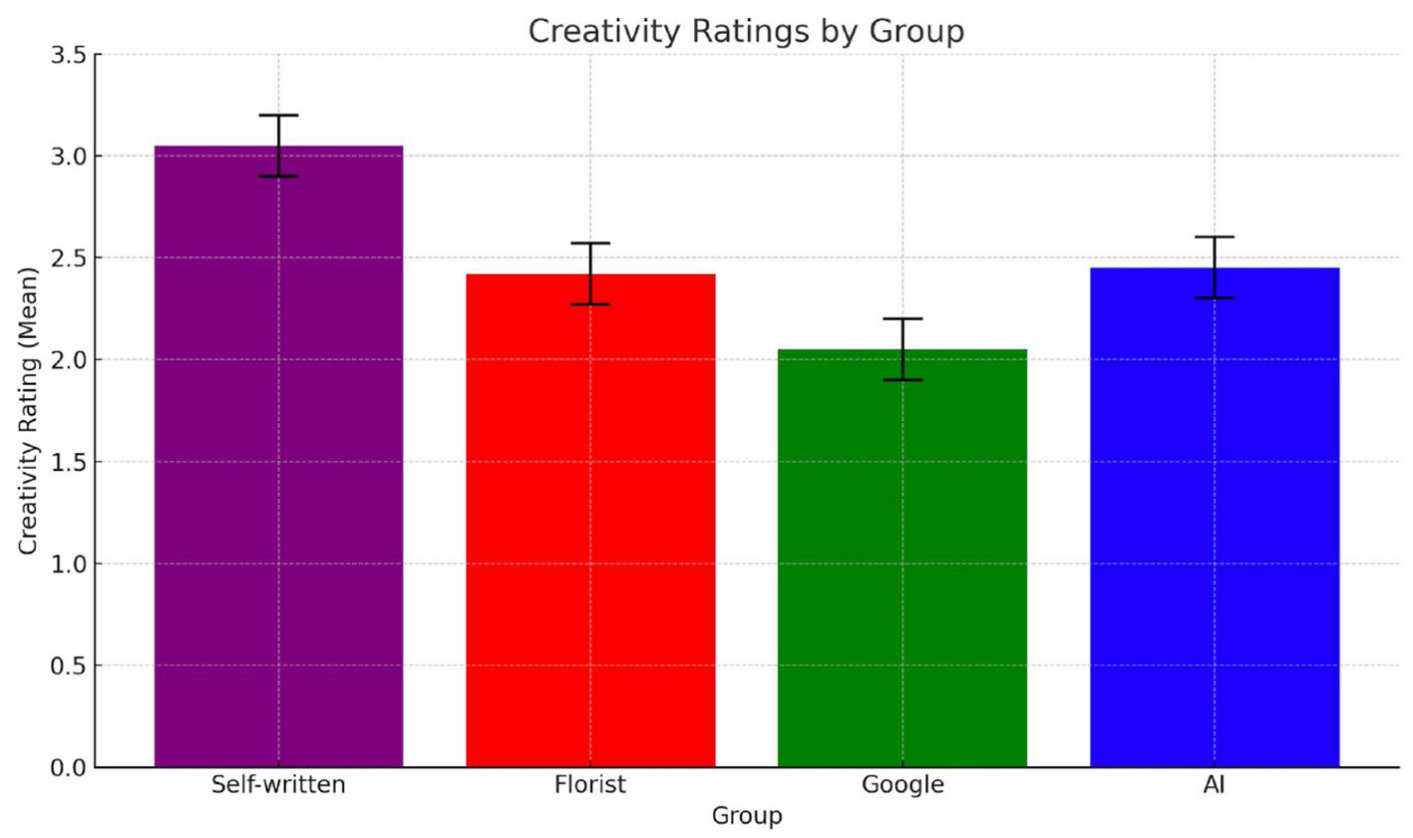
Mean creativity ratings across the four groups evaluating the card written by different sources: self-written by a friend (purple); written by a florist (red); found on Google (green); AI-generated (blue).

**Figure 5:**
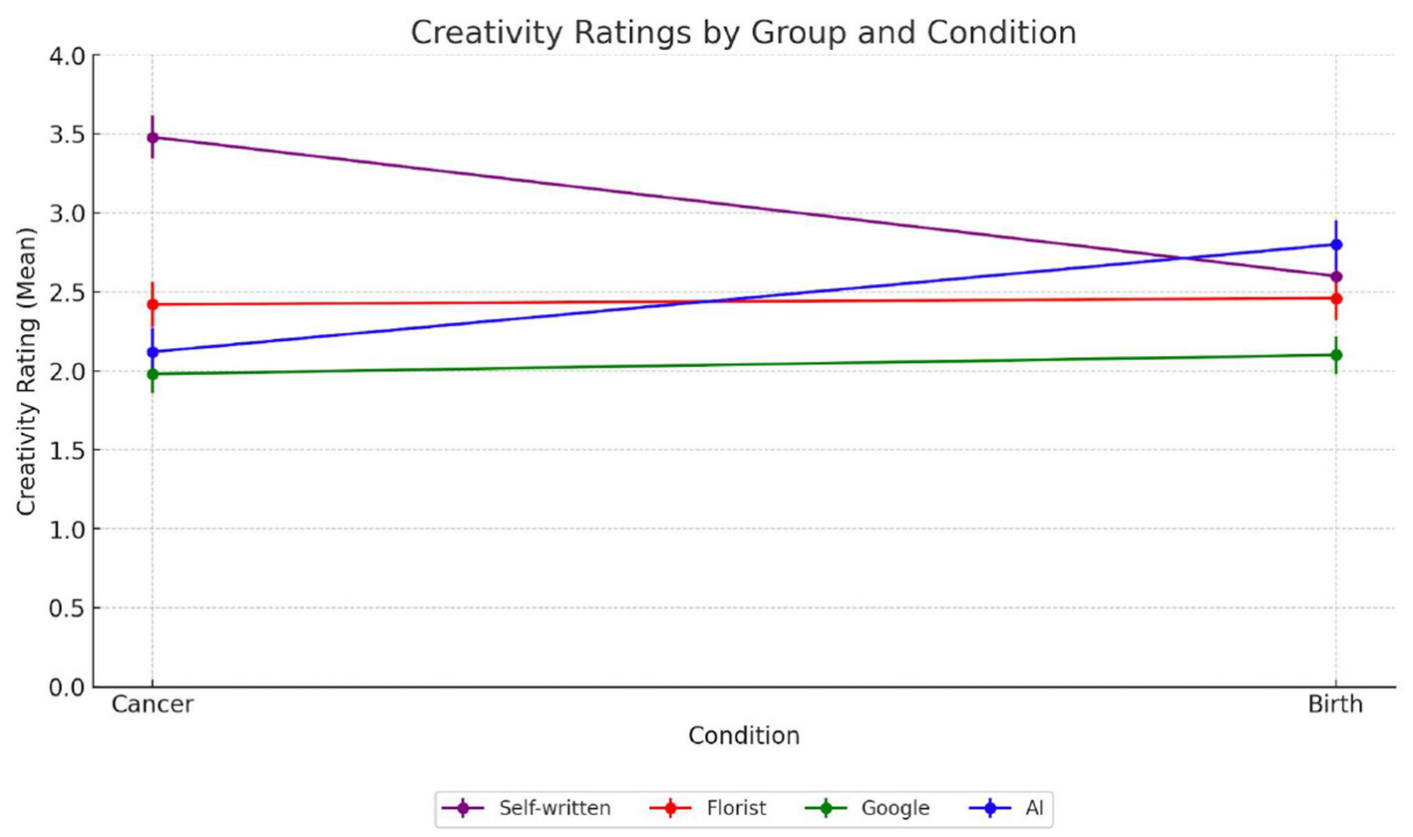
Average creativity ratings across the four groups evaluating the note written by different sources in the serious illness and birth condition: self-written by a friend (purple); written by a florist (red); found on Google (green); AI-generated (blue).

Creativity, when situated in affect-laden contexts, is not judged solely on formal or stylistic criteria but is often imbued with assumptions about the sender's emotional presence and symbolic effort. In Study 2, creativity ratings differed significantly across source conditions, F_(3, 156)_ = 5.77, *p* = 0.0009. As shown in Figure 4, messages attributed to a close friend were rated as significantly more creative (M = 3.05, SD = 0.87) than those from all other sources. This suggests that emotional proximity enhances perceived originality, likely because participants infer personal investment, symbolic resonance and contextual sensitivity. We use the term “symbolic resonance” to describe the feeling that the form, content, and source of a message are coherent and “resonate” with the cultural and relational codes of the recipient. By contrast, messages attributed to AI (M = 1.68, SD = 0.98), the florist (M = 2.44, SD = 0.89), and Google (M = 2.21, SD = 0.76) received significantly lower ratings. *Post hoc* comparisons confirmed that each of these sources was perceived as less creative than the close friend (*p* < 0.01 for all comparisons), but did not differ significantly from one another. This clustering suggests that once emotional proximity is removed—whether due to artificiality or relational unfamiliarity– participants are less inclined to interpret the message as symbolically novel or expressive. Importantly, these effects were moderated by emotional context. As shown in Figure 5, creativity ratings in the terminal illness scenario were significantly higher when the sender was a close friend compared to the childbirth scenario, F_(1, 78)_ = 4.59, *p* = 0.039. In the terminal illness condition, the emotional gravity of the situation appears to heighten sensitivity to relational cues, magnifying the expressive value of communication from emotionally close sources. By contrast, in the childbirth condition, creativity ratings did not differ significantly across source conditions, indicating that celebratory contexts may neutralize attributional effects by lowering the normative demand for symbolic depth. These findings support the hypothesis that Hypothesis 2a according to which creativity is perceived as highest when authorship is linked to a relationally close source, particularly in emotionally vulnerable contexts. Once proximity is removed, whether through artificiality or anonymity, creativity judgments converge toward lower and statistically indistinct levels.

#### 4.2.2 Perceived authenticity

**Figure 6:**
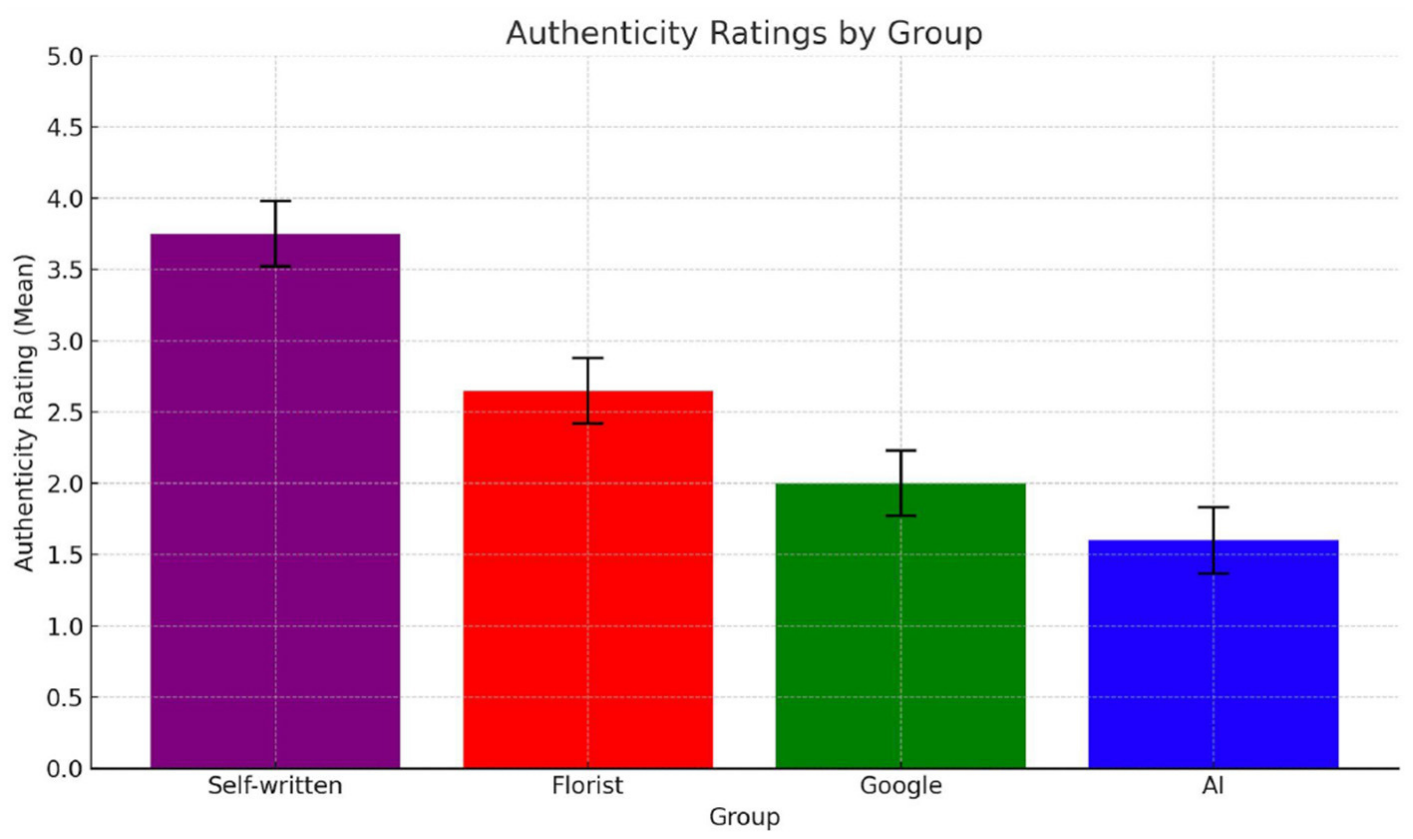
Mean authenticity ratings across the four groups evaluating the card written by different sources: self-written by a friend (purple); written by a florist (red); found on Google (green); AI-generated (blue).

**Figure 7:**
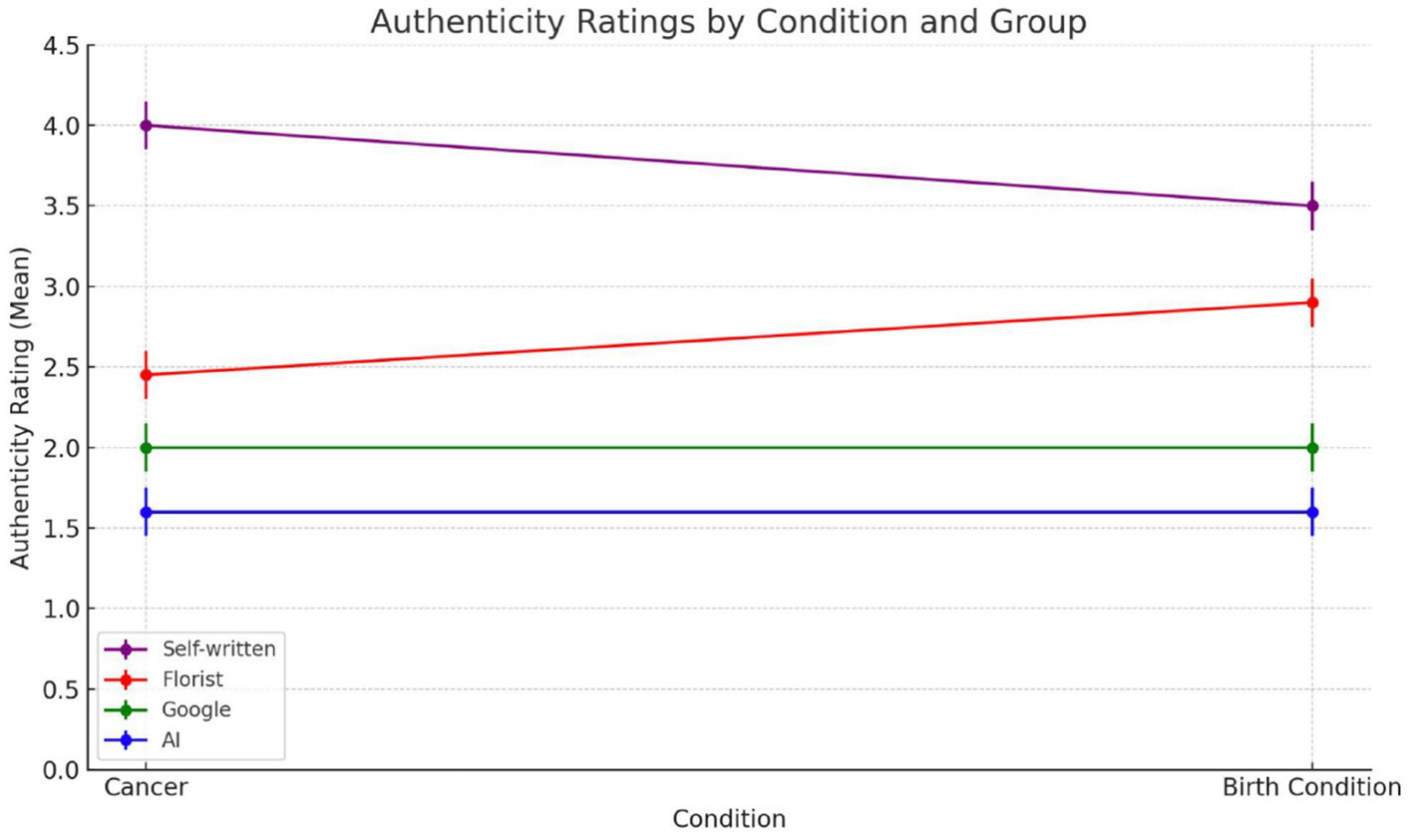
Average authenticity ratings across the four groups evaluating the note written by different sources: self-written by a friend (purple); written by a florist (red); found on Google (green); AI-generated (blue).

Authenticity, conceptualized as the congruence between emotional intention and expression, is especially salient in interpersonal communication where sincerity and relational investment are expected. Results from Study 2 show that authenticity evaluations were strongly influenced by source attribution, *F*_(3, 156)_ = 57.27, *p* < 0.001. As shown in Figure 6, messages attributed to a close friend received the highest authenticity ratings (M = 3.78, SD = 1.07), significantly exceeding those attributed to AI (M = 2.08, SD = 1.33), the florist (M = 2.35, SD = 1.15), and Google (M = 2.11, SD = 0.93). All comparisons between the friend and other sources were statistically significant (*p* < 0.001), whereas no significant differences emerged among AI, florist, and Google conditions. These results confirm that authenticity is not merely a linguistic property, but an attributional inference grounded in relational proximity and presumed intentionality. When disaggregated by emotional context (Figure 7), attributional effects remained robust. In the **t**erminal illness condition, the friend was rated as significantly more authentic than all other sources, and notably, even the florist—a human but relationally neutral source—was rated as more authentic than AI (*p* = 0.0011). This suggests that human authorship can modestly elevate authenticity, but only relational closeness meaningfully validates it. In the childbirth context, the friend again outperformed AI and Google in authenticity ratings. However, unlike in the illness scenario, the florist condition approached the friend in perceived authenticity and significantly exceeded both AI and Google. These results imply that in less emotionally fraught scenarios, judgments of sincerity may be more elastic: while relational proximity remains influential, human authorship—even from distant actors—is perceived as more credible than artificial or institutional sources. Taken together, these findings support Hypothesis 2 in which relational closeness reliably enhances perceived authenticity across contexts, but emotional intensity moderates the degree to which attribution to human or institutional senders influences evaluations.

#### 4.2.3 Perceived moral respect

**Figure 8:**
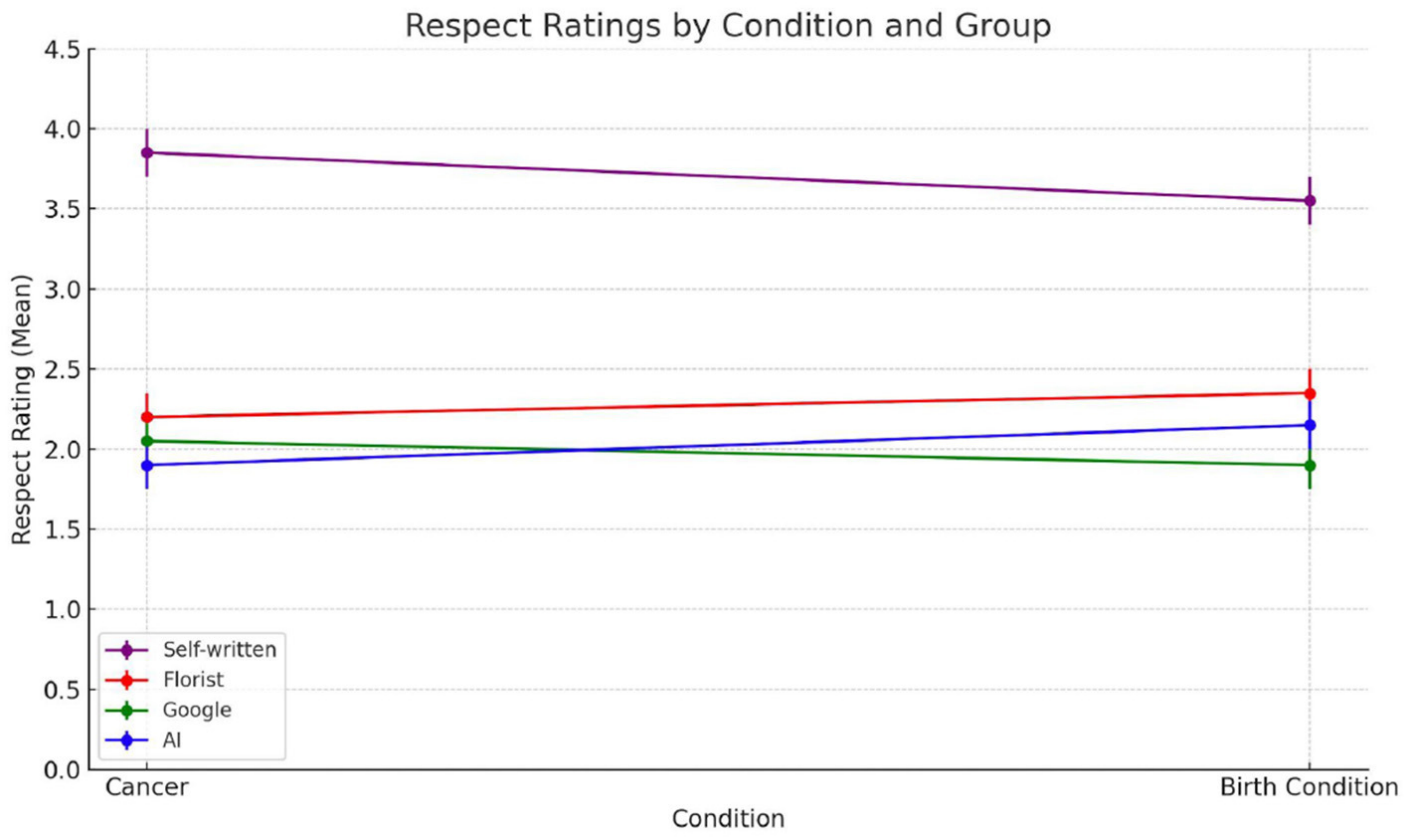
Average respect ratings across the four groups evaluating the note written by different sources: self-written by a friend (purple); written by a florist (red); found on Google (green); AI-generated (blue).

Respect, in emotionally significant communication, is more than politeness—it conveys moral recognition, attentiveness, and the legitimacy of the sender's engagement. Consistent with this understanding, source attribution had a significant effect on respect ratings, F_(3, 156)_ = 32.66, *p* < 0.001 (Figure 8). Messages attributed to a close friend were rated as the most respectful (M = 4.11, SD = 1.02), significantly higher than those attributed to AI (M = 2.44, SD = 1.19), the florist (M = 2.72, SD = 1.06), and Google (M = 2.39, SD = 0.98). All pairwise comparisons between the friend and other sources were statistically significant (*p* < 0.001). No significant differences were observed among AI, florist, and Google, indicating that when emotional proximity is absent, the symbolic capacity to convey respect diminishes uniformly—regardless of whether the source is human, institutional, or artificial. This pattern held consistently across both emotional contexts. In the terminal illness condition, the close friend was perceived as significantly more respectful than AI, florist, or Google (*p* < 0.001 in all comparisons), with no significant differences among the three latter sources. Surprisingly, the same structure appeared in the childbirth context. Even in a celebratory scenario, relational closeness remained the key differentiator in respect evaluations, while distant or artificial sources were rated similarly and substantially lower. These findings provide strong support for Hypothesis 2, in which unlike creativity, which was modulated by emotional context, and authenticity, which permitted more nuanced evaluations, respect emerged as the most robustly relational construct. Across all conditions, only attribution to a close human source conveyed the symbolic and moral gravity expected in high-stakes communication.

## 5 Discussion

Emotionally significant communication is more than a transmission of information—it is a symbolic act of relational and moral meaning. This study shows that perceptions of creativity, authenticity, and moral respect in such communication are deeply shaped by the attribution of message source and the perceived effort embedded in the medium. Across both experiments, messages believed to originate from emotionally close sources—especially friends—were consistently evaluated more favorably than those attributed to strangers or AI systems. These findings underscore that judgments are not reducible to message content or linguistic sophistication, but are co-constructed through contextual cues and social expectations (Van Boven et al., 2003; Erickson, 1995).

This attributional asymmetry is particularly salient in emotionally charged contexts. Participants in the terminal illness condition evaluated AI-generated messages more harshly than those in the childbirth condition, suggesting that moral and symbolic expectations intensify when emotional stakes are high. In such moments, communication is not only expected to be correct—it is expected to be caring. Our findings align with prior work suggesting that AI lacks the perceived intentionality, emotional investment, and moral presence necessary to satisfy these expectations (Guzman and Lewis, 2020; Sundar and Lee, 2022).

Furthermore, we introduced the concept of an emotional proximity scale to capture how attributional distance—moving from self to friend, stranger, and finally to AI—shapes evaluative outcomes. This scale reflects not just psychological closeness but symbolic legitimacy. Messages from friends embody care and intentionality; those from AI, despite often being linguistically competent, are seen as emotionally detached and morally inadequate. These results support Hypotheses 1 and 1a, confirming that source identity plays a foundational role in meaning construction. The largest downward reappraisals occurred when messages initially perceived as touching were later revealed to be authored by AI—a pattern consistent with Hypothesis 1b and with attribution-based models of symbolic dissonance (Ovsyannikova et al., 2025; Złotowski et al., 2017).

This re-evaluation dynamic is further influenced by conversational pragmatics. According to Grice (1975) maxim of relevance, communicative acts are assumed to carry meaning not only through their literal content but through contextual inferences. Revealing the message source after initial exposure likely signaled to participants that the source was a meaningful feature, prompting reassessment. This pragmatic effect reveals how human judgments respond to symbolic coherence or its violation—a finding that complements theories of affective and moral appraisal in communication (Tyler and Blader, 2003; Harter, 2002).

An alternative interpretation of our findings draws from theories of textual reception and psychological projection. While we originally framed the results as evidence of the limitations of AI in conveying emotional depth compared to human communication, it is also possible that participants' evaluations were shaped not by differences in the message itself, which remained constant across conditions, but by extratextual knowledge of who authored it. In this view, the message's perceived qualities (e.g., creativity or respect) are not intrinsic to the text, but constructed through expectations and relational context. For example, the observed increase in creativity ratings upon learning that Roberta wrote the message may reflect a form of compensatory projection, where participants attribute greater symbolic intentionality or emotional effort to human senders—especially close others—based on internalized beliefs about human communicative superiority. This view resonates with literary theory, which emphasizes the role of authorship and framing in shaping textual meaning, and with psychoanalytic perspectives that highlight how individuals unconsciously ascribe desirable traits (such as depth, care, or originality) to messages authored by emotionally significant humans. This interpretive lens underscores the flexibility—and subjectivity—of meaning-making in emotionally charged communication, especially when source information is introduced after initial exposure.

Crucially, the presence of anthropomorphic cues appears to both enable and destabilize emotional resonance. AI systems that use emotionally expressive language or personalized tone may initially appear creative or sincere, but this perception collapses once users learn the message is artificial (Epley et al., 2007; Waytz et al., 2010). This paradox reflects the “authenticity valley”, where near-human expressiveness evokes discomfort rather than connection when dissonance between form and source becomes apparent (Mori et al., 2012; Nass and Moon, 2000). The very features that enhance plausibility in human-like AI also heighten expectations—and when those expectations are unmet, users experience emotional and moral incongruity (Złotowski et al., 2017).

The medium of communication amplified these dynamics. Handwritten notes consistently outperformed typed messages across all conditions, even when the text content remained identical. This finding reinforces that symbolic value is derived not only from what is said, but how it is conveyed. Handwriting signals personal effort and emotional investment—qualities closely tied to authenticity and respect (Rozin and Nemeroff, 1994; Bickmore et al., 2009). In contrast, typed or AI-generated texts were seen as efficient but emotionally thin, particularly when emotional resonance and moral salience were expected. This challenges some of the foundational assumptions of the CASA paradigm (Computers Are Social Actors; Reeves and Nass, 1996), which posits that people instinctively respond to media technologies as if they were human. Our results suggest a more nuanced reality: while users may anthropomorphize machines in neutral or utilitarian contexts, when moral recognition, symbolic legitimacy, and emotional intimacy are at stake, the artificial nature of the source becomes salient and even disqualifying. In these high-stakes moments, it is not enough for a message to be coherent or competent; it must be embodied by an agent perceived as capable of moral agency and symbolic reciprocity. This marks a threshold where the “as if” of CASA gives way to a demand for genuine relational presence.

## 6 Implications

This study contributes to theory in several meaningful ways. First, it advances attribution theory by showing that perceptions of creativity, authenticity, and moral respect are constructed not simply through linguistic cues, but through inferences about the sender's identity, intentionality, and emotional proximity. In emotionally significant communication, the *who* is just as important as the *what*. Second, the findings offer a refinement of anthropomorphism theory. While prior research has highlighted how human-like features can increase trust or engagement, our results suggest that anthropomorphism raises normative expectations that, if unmet, provoke stronger backlash. This supports the emerging notion of the “authenticity valley”, in which systems that appear nearly human invite moral standards they cannot meet—resulting in evaluative disruption when artificiality is revealed. Third, we offer a conceptual bridge between relational communication theory and affective computing. Our emotional proximity scale positions attributional distance as a core symbolic variable, helping explain why some messages are perceived as emotionally resonant and others as hollow, even when their surface content is identical.

Furthermore, this study also carries significant implications for the design and deployment of AI systems in emotionally sensitive domains. Organizations should exercise caution when using AI to generate messages related to grief, celebration, or caregiving. While AI-generated content can be stylistically fluent and contextually relevant, its lack of perceived emotional intentionality and moral presence may lead to feelings of inauthenticity or disrespect when the sender's identity is revealed. Moreover, designers of emotionally intelligent systems should recognize that anthropomorphic features are a double-edged sword. While they may enhance initial engagement, they also raise the stakes of symbolic coherence—inviting deeper disappointment when users perceive a mismatch between form and source. A further practical implication concerns message transparency and framing. If AI is to be used in emotionally significant communication, it may be advisable to pair it with human oversight, disclose authorship clearly, or frame the message as co-authored with a trusted human. These strategies may help mitigate symbolic dissonance and reinforce the message's emotional legitimacy.

## 7 Limitations and future research

While this study offers valuable insights, stemming from its intrinsic limitations and the findings, we propose several areas for further exploration. First, longitudinal studies could examine how perceptions of AI-authored communication evolve over time, particularly as users grow more familiar with generative technologies. Delayed dissonance or normalization effects could reshape how emotional and moral meaning is constructed. Second, future work could explore how the emotional proximity scale operates across different domains (e.g., such as education, customer service, or organizational leadership) where symbolic legitimacy and perceived effort also play critical roles. Third, the effects of cultural variation warrant attention. Norms around authenticity, creativity, and respect differ across societies, and future studies could test whether the attributional patterns observed here hold across collectivist vs. individualist cultures, or high-context vs. low-context communication environments. Fourth, future research should incorporate individual-level moderators, including technological literacy, emotional intelligence, and relational orientation. These traits may mediate how individuals interpret and evaluate AI-generated messages. Given the limited sample size and exploratory nature of Study 2, its findings should be interpreted with caution. While the between-group design offers useful preliminary insights, it requires replication with larger, pre-registered samples to confirm its robustness. Further studies are needed to replicate and extend these findings using larger and more diverse samples. Future research should examine the reliability of source-based attribution effects across cultures, message contexts, and emotionally salient scenarios. Our findings were obtained within a Western, individualist cultural context, where authenticity and emotional resonance are often tied to personal expression and perceived autonomy. However, in collectivist cultures, authenticity may be more closely linked to harmony, relational duty, or conformity to social expectations. These cultural differences may shape how people interpret emotionally supportive messages, especially when the sender is an AI or a socially distant actor. We encourage future cross-cultural research to examine whether the results replicate across diverse cultural contexts, and how local norms influence perceptions of sincerity, appropriateness, and moral alignment. Lastly, expanding the methodological scope to include multimodal or embodied communication (e.g., video, voice, or avatars) could shed light on how sensory richness and social presence interact with attributional cues to shape perceptions of authenticity and moral worth.

## 8 Conclusion

This study demonstrates the critical influence of message source and medium on how emotionally significant communication is perceived. Messages attributed to a close friend were consistently rated higher in creativity, authenticity, and moral respect than those associated with a stranger or an artificial intelligence system. These findings highlight the central role of emotional proximity and perceived effort in shaping the symbolic and affective value of communication. The introduction of the *emotional proximity scale* offers a conceptual tool to capture the gradations of emotional engagement inferred from the sender's identity, enriching our understanding of how relational closeness modulates emotional resonance.

Despite their fluency and contextual relevance, AI-generated messages were broadly perceived as emotionally detached and symbolically hollow—particularly in the terminal illness scenario, where expectations for sincerity and moral presence were most pronounced. This reflects a core limitation of current AI: its inability to convey the care, intentionality, and relational investment that characterize human expressions of empathy. While AI can convincingly simulate affective tone, its lack of experiential subjectivity and moral agency often leads to diminished perceptions of authenticity and respect once its artificial nature is revealed. These results align with broader literature on anthropomorphism and attribution, supporting the view that users interpret emotionally charged messages through a dual lens: the linguistic quality of the content and the presumed moral identity of the sender.

Our findings raise important ethical and design considerations for integrating AI into emotionally sensitive domains. While AI systems offer benefits in consistency, scalability, and personalization, their deployment in contexts that require symbolic depth and emotional sincerity must be approached with caution. Design strategies that emphasize human oversight, clearly communicate the nature of AI involvement, or embed anthropomorphic cues calibrated to the context may help mitigate the perception of emotional detachment. However, as our results suggest, such strategies also risk triggering attributional dissonance if the AI fails to meet the heightened normative expectations it invites. In this light, the symbolic coherence between content, context, and sender identity becomes a crucial benchmark for evaluating the appropriateness of AI-mediated communication.

By theorizing how attributional processes and anthropomorphic design shape perceptions of creativity, authenticity, and moral respect, this research contributes to ongoing conversations in human-machine communication, affective computing, and the moral psychology of artificial agents. It provides both conceptual and practical guidance for designing AI systems that operate not only with technical fluency but also with symbolic and emotional sensitivity. As AI continues to permeate interpersonal and emotionally meaningful domains, understanding its interpretive boundaries and social expectations will be essential for fostering more ethical, respectful, and resonant human-AI interactions.

## Data Availability

The raw data supporting the conclusions of this article will be made available by the authors, without undue reservation.
